# Publisher Correction: Author Correction: A patient-centric dataset of images and metadata for identifying melanomas using clinical context

**DOI:** 10.1038/s41597-021-00879-x

**Published:** 2021-03-16

**Authors:** Veronica Rotemberg, Nicholas Kurtansky, Brigid Betz-Stablein, Liam Caffery, Emmanouil Chousakos, Noel Codella, Marc Combalia, Stephen Dusza, Pascale Guitera, David Gutman, Allan Halpern, Brian Helba, Harald Kittler, Kivanc Kose, Steve Langer, Konstantinos Lioprys, Josep Malvehy, Shenara Musthaq, Jabpani Nanda, Ofer Reiter, George Shih, Alexander Stratigos, Philipp Tschandl, Jochen Weber, H. Peter Soyer

**Affiliations:** 1 Dermatology Service, Department of Medicine, Memorial Sloan Kettering Cancer Center, New York, NY USA; 2 The University of Queensland Diamantina Institute, The University of Queensland, Dermatology Research Centre, Brisbane, Australia; 3 University of Athens Medical School, Athens, Greece; 4 Microsoft, Redmond, WA USA; 5 Melanoma Unit, Dermatology Department, Hospital Cĺınic Barcelona, Universitat De Barcelona, IDIBAPS, Barcelona, Spain; 6 Melanoma Institute Australia and Sydney Melanoma Diagnostic Center, Sydney, Australia; 7 Emory University School of Medicine, Department of Biomedical Informatics, Atlanta, GA USA; 8 Kitware, Inc., Clifton Park, NY USA; 9 Medical University of Vienna, Department of Dermatology, Vienna, Austria; 10 Division of Radiology Informatics, Department of Radiology, Mayo Clinic, Rochester, MN USA; 11 SUNY Downstate Medical School, New York, NY USA; 12 Stony Brook Medical School, Stony Brook, NY USA; 13 Rabin Medical Center, Tel Aviv, Israel; 14 Department of Radiology, Weill Cornell Medical College, New York, NY USA

**Keywords:** Skin cancer, Skin manifestations

Correction to: *Scientific Data* 10.1038/s41597-021-00865-3, published online 05 March 2021

In the corrected version of this Data Descriptor, an error was introduced in the spelling of H. Peter Soyer which was incorrectly given as H. Peter Seter. This has now been corrected in both the PDF and HTML versions of the Data Descriptor.

